# End-to-End Deep Image Reconstruction From Human Brain Activity

**DOI:** 10.3389/fncom.2019.00021

**Published:** 2019-04-12

**Authors:** Guohua Shen, Kshitij Dwivedi, Kei Majima, Tomoyasu Horikawa, Yukiyasu Kamitani

**Affiliations:** ^1^Computational Neuroscience Laboratories, Advanced Telecommunications Research Institute International, Kyoto, Japan; ^2^Graduate School of Informatics, Kyoto University, Kyoto, Japan

**Keywords:** brain decoding, visual image reconstruction, functional magnetic resonance imaging, deep neural networks, generative adversarial networks

## Abstract

Deep neural networks (DNNs) have recently been applied successfully to brain decoding and image reconstruction from functional magnetic resonance imaging (fMRI) activity. However, direct training of a DNN with fMRI data is often avoided because the size of available data is thought to be insufficient for training a complex network with numerous parameters. Instead, a pre-trained DNN usually serves as a proxy for hierarchical visual representations, and fMRI data are used to decode individual DNN features of a stimulus image using a simple linear model, which are then passed to a reconstruction module. Here, we directly trained a DNN model with fMRI data and the corresponding stimulus images to build an end-to-end reconstruction model. We accomplished this by training a generative adversarial network with an additional loss term that was defined in high-level feature space (feature loss) using up to 6,000 training data samples (natural images and fMRI responses). The above model was tested on independent datasets and directly reconstructed image using an fMRI pattern as the input. Reconstructions obtained from our proposed method resembled the test stimuli (natural and artificial images) and reconstruction accuracy increased as a function of training-data size. Ablation analyses indicated that the feature loss that we employed played a critical role in achieving accurate reconstruction. Our results show that the end-to-end model can learn a direct mapping between brain activity and perception.

## Introduction

Advances in the deep learning have opened new directions to decode and visualize the information present in the human brain. In the past few years, deep neural networks (DNNs) have been successfully used to reconstruct visual content from brain activity measured by functional magnetic resonance imaging (fMRI) (Güçlütürk et al., 2017; Han et al., 2017; Seeliger et al., 2018; Shen et al., 2019).

The reconstruction studies avoid training a DNN model directly on the fMRI data because of limited dataset size in fMRI studies. To solve the limited dataset size issue, the feature representation from a DNN pre-trained on a large scale image dataset is usually used as a proxy for the neural representations of the human visual system. Hence, these decoded-feature-based methods involve two independent steps, (1) decoding DNN features from fMRI activity and (2) reconstruction using the decoded DNN features.

Different from fMRI studies, DNNs in computer vision for image generation are usually trained in the end-to-end manner with large datasets. For instance, Mansimov et al. (2015) trained a caption-to-image model on Microsoft COCO dataset that consists of 82,783 images, each annotated with at least 5 captions. Dosovitskiy and Brox (2016a) trained a DNN model on ImageNet training dataset (over 1.2 million images) to reconstruct images from corresponding DNN features. Due to availability of large-scale image datasets, the above image-generation studies can train DNNs using an end-to-end approach to directly generate images from a modality correlated with the images. In contrast, the largest fMRI dataset used for reconstruction in Shen et al. (2019) consisted of only 6,000 training samples. Thus, training a DNN to reconstruct images directly from fMRI data is often avoided and considered infeasible because of the smaller datasets.

Learning a direct mapping between brain activity and perception of the outside world or subjective experiences would be advantageous over the previous decoded-feature-based methods due to the following reason. Decoding features from fMRI is constrained by the pre-trained DNN features which were optimized in a prior without brain data that may not be optimal for decoding them from brain activity. Therefore, information loss occurs in the decoding process which affects the quality of reconstruction. A direct mapping can help in reducing the information loss mentioned above.

In this study, we sought to evaluate the potential of the end-to-end approach for directly mapping fMRI activity to stimulus space given a limited training dataset. In the end-to-end approach, the input to the DNN is the fMRI activity and the output of the DNN is the reconstruction of the perceived stimulus. If reconstruction using the end-to-end approach is successful, we can avoid the feature-decoding step (step 1 above) that leads to information loss.

For designing an end-to-end DNN model to reconstruct images from fMRI data, we considered the models that transform image representations such as DNN features to original image as the potential candidates. The motivation behind this is that the fMRI activity is the neural representation of the perceived image and thus can be considered as an image representation. Also, in previous studies (Agrawal et al., 2014; Khaligh-Razavi and Kriegeskorte, 2014; Güçlü and van Gerven, 2015a,b; Cichy et al., 2016; Horikawa and Kamitani, 2017) fMRI activity has already been shown to be correlated to DNN features. Therefore, for this study, we adopted the model from Dosovitskiy and Brox (2016b) to reconstruct the image from fMRI activity.

For the end-to-end image reconstruction model used in this study, the model needs to be optimized with suitable choice of loss functions relevant to our problem. Dosovitskiy and Brox (2016a) first proposed a DNN-based method for reconstructing original images from their corresponding features by optimization within image space. Loss in image space usually results in poor reconstruction because it generates an average of all possible reconstructions having the same distance in image space, and hence the reconstructed images are blurred. The feature loss in high dimensional space, also called perceptual loss, constrains the reconstruction to be perceptually similar to the original image. Adversarial loss (Goodfellow et al., 2014) constrains the distribution of the reconstructed images to be close to the distribution of natural images. In a subsequent study, Dosovitskiy and Brox (2016b) have also showed that reconstruction from features is improved by introducing feature and adversarial loss terms. Thus, we adopted this latter approach for reconstructing perceived stimuli directly from the fMRI activity. Specifically, we modified their model to take input directly from the fMRI activity and trained the model from scratch with the dataset from Shen et al. (2019).

Here, we present a novel approach to visualize perceptual content from human brain activity by an end-to-end deep image reconstruction model which can directly map fMRI activity in the visual cortex to stimuli observed during perception. Our end-to-end deep image reconstruction model was accomplished by directly training a deep generative adversarial network with a perceptual loss term with fMRI data and the corresponding stimulus images. We demonstrated that the reconstructions obtained from our proposed method resembled the original stimulus images. We further explored the generalizability of our reconstruction model (trained solely with natural images and fMRI responses) to artificial images. To understand the effect of training-dataset size on reconstruction quality, we compared reconstruction results across gradually increasing dataset sizes (from 120 to 6,000 samples). Finally, to investigate the effects of different loss functions used in the reconstruction, we performed an ablation study that objectively and subjectively compared reconstruction results as loss functions were removed one at a time.

## Materials and Methods

In this section, we briefly describe the methods we used for our experiments and the details of the dataset. For more details regarding image reconstruction, please refer to Dosovitskiy and Brox (2016b), and for details regarding the dataset, please refer to Shen et al. (2019).

### Problem Statement

Let **x**∈**R**^*w*×*h*×3^ be the stimulus image displayed in the perception experiment, where *w* and *h* are width and height of the stimulus image and 3 denotes the number of channels (RGB) of the color image. Let **v**∈ℝ^*D*^ be the corresponding preprocessed fMRI vector for the brain activity recorded during the perception of the image, with *D* being the number of voxels in the visual cortex (VC). We are interested in obtaining a reconstruction of the stimulus from fMRI vector **v**.

To solve this problem, we use a DNN **G**_**θ**_ with parameters **θ**, which performs non-linear operations on **v** to obtain a plausible reconstruction **G**_**θ**_(**v**) of the stimulus image.

### Image Reconstruction Model

To reconstruct stimulus images from fMRI data, we modified the DNN model proposed by Dosovitskiy and Brox (2016b).

**Figure 1:**
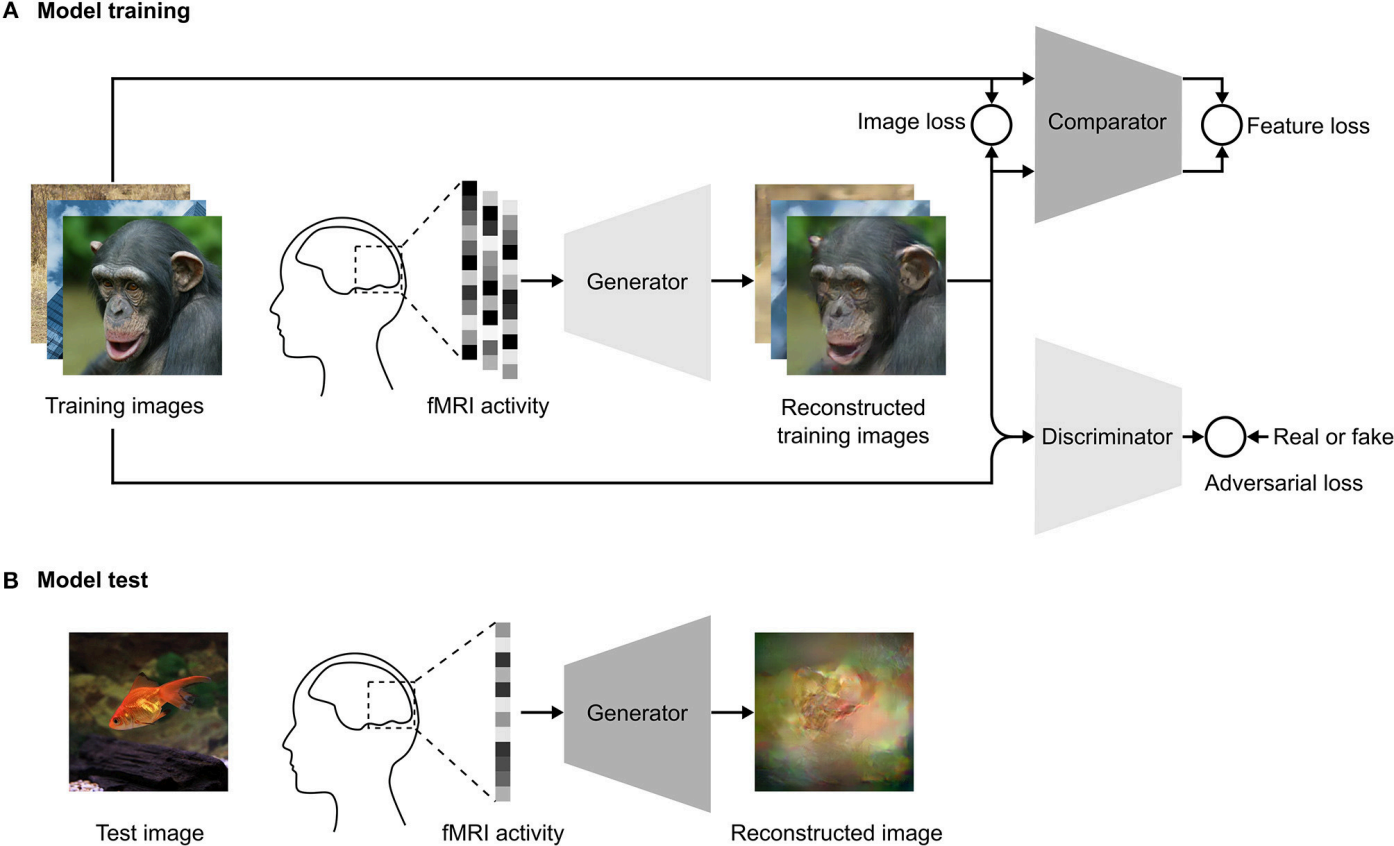
Schematics of our reconstruction approach. **(A)** Model training. We use an adversarial training strategy adopted from Dosovitskiy and Brox (2016b), which consists of three DNNs: a generator, a comparator, and a discriminator. The training images are presented to a human subject while brain activity is measured by fMRI. The fMRI activity is used as input to the generator. The generator is trained to reconstruct the images from the fMRI activity to be as similar as possible to the presented training images in both pixel and feature space. The adversarial loss constrains the generator so that reconstructed images fool the discriminator into classifying them as the true training images. The discriminator is trained to distinguish between the reconstructed image and the true training image. The comparator is a pre-trained DNN that was trained to recognize objects in natural images. Both the reconstructed and true training images are used as input to the comparator, which compares the image similarity in feature space. **(B)** Model test. In the test phase, the images are reconstructed by providing the fMRI activity associated with the test image as the input to the generator.

For each fMRI data vector **v** corresponding to a stimulus image **x**, the model was trained to generate a plausible reconstructed image **G**_**θ**_**(v)**. In the training step, the network architecture (Figure 1A) consisted of three convolutional neural networks: a generator **G**_**θ**_ that transformed the fMRI vector **v** to **G**_**θ**_**(v)**, a discriminator **D**_**Φ**_ that discriminated the reconstructed image **G**_**θ**_**(v)** from the natural image **x**, and a comparator **C** that compared the reconstructed image **G**_**θ**_**(v)** with the original stimulus image **x** in feature space. During test time, we only need the generator (Figure 1B) to reconstruct images from fMRI data.

The input to the generator was the fMRI vector **v** from the VC and the output was the reconstructed image **G**_**θ**_**(*v*)**. The generator consisted of three fully connected layers followed by six upconvolution layers that generated the final reconstruction image **G**_**θ**_**(*v*)**. The comparator network **C** was Caffenet (Krizhevsky et al., 2012), which was trained on the ImageNet dataset for the image classification task. The Caffenet model is a replication of the Alexnet model with the order of pooling and normalization layers switched and without relighting data-augmentation during training. The network consisted of five convolutional and three fully connected layers. We used the last convolutional layer of the comparator to compare the reconstructed image with the original image in feature space. The parameters of the comparator were not updated during training of the reconstruction model.

The discriminator **D**_**Φ**_ consisted of five convolutional layers followed by an average pooling layer and two fully connected layers. The output layer of the discriminator was a 2-way softmax and the network was trained to discriminate the reconstructed image from the original image. The generator was trained concurrently to optimize the adversarial loss function, which fooled the discriminator into classifying the reconstructed image as the real stimulus image. The adversarial loss forces the generator to generate more realistic images that are closer to the image distribution of the training data.

The generator was modified to take its input from fMRI data instead of DNN features. The discriminator in Dosovitskiy and Brox (2016b) was provided two inputs, the image and corresponding feature from the comparator, however, we modified the discriminator to receive only the image as the input. The architecture of the comparator network was the same as in Dosovitskiy and Brox (2016b).

Let **X**_*i*_ denote the *i* th stimulus image in the dataset, **V**_*i*_ denote the corresponding fMRI data, and **G**_**θ**_**(V**_*i*_**)** denote the corresponding reconstructed output image of the generator. The generator **G**_**θ**_ had parameters **θ**, which were updated to minimize the weighted sum of three loss terms for a mini-batch that used stochastic gradient descent: loss in image space *L*_img_, feature loss *L*_feat_, and adversarial loss *L*_adv_:

$$L(\theta ,\Phi )=\lambda_{\text{img}}L_{\text{img}}(\theta )+\;\lambda_{\text{feat}}L_{\text{feat}}(\theta )+\;\lambda_{\text{adv}}L_{\text{adv}}(\theta ,\Phi )$$

where

$$\begin{array}{l} L_{\text{img}}(\theta )=\;\sum_{i}∥G_{\theta }(V_{i})-X_{i}{∥}_{2}^{2}\\ L_{\text{feat}}(\theta )=\sum_{i}∥C\left(G_{\theta }(V_{i})\right)-C\left(X_{i}\right){∥}_{2}^{2}\;\\ L_{\text{adv}}(\theta ,\Phi )=\;-\sum_{i}\text{log }D_{\Phi }(G_{\theta }(V_{i})) \end{array}$$

and λ_img_, λ_feat_, and λ_adv_ denote the weights of the loss in image space *L*_img_, feature loss *L*_feat_, and adversarial loss *L*_adv_, respectively.

The discriminator was trained concurrently with the generator to minimize *L*_discr_:

$$L_{\text{discr}}\left(\Phi \right)=\;-\sum_{i}^{}\text{log}\left(D_{\Phi }\left(X_{i}\right)\right)+\text{log}\left(1-D_{\Phi }\left(G_{\theta }\left(V_{i}\right)\right)\right).$$

The parameters of the comparator **C** were fixed throughout the training because it was only used for the comparison in feature space, and thus did not require any update.

We trained the system using the Caffe framework (Jia et al., 2014) and modified the implementation of the model provided by Dosovitskiy and Brox (2016b). The weights of the generator and discriminator were initialized using MSRA (He et al., 2015) initialization. The comparator weights were initialized by Caffenet weights trained on ImageNet classification. We used Adam solver (Kingma and Ba, 2015) with momentum β_1_ = 0.9, β_2_ = 0.999 and an initial learning rate 0.0002 for optimization. We used a batch size of 64 and trained for 500,000 mini-batch iterations in all experiments. Following this training procedure similar to Dosovitskiy and Brox (2016b), we temporarily stopped updating the discriminator if the ratio of *L*_discr_ to *L*_adv_ was below 0.1. This was done to prevent the discriminator from overfitting. The weights of the individual loss functions λ_img_, λ_feat_, and λ_adv_ were set to $\lambda_{\text{img}}={2\times 10}^{6}$, λ_feat_ = 0.01, and λ_adv_ = 100.

We applied image jittering during the training for data augmentation and to take into account subject's eye movement during the image presentation experiment. Generally, eye movement was approximately one degree of visual angle for a typical subject. The viewing angle for the stimulus images was 12°. All training images were resized to 248 × 248 pixels before training. During training, we randomly cropped a 227 × 227 pixel window from each training image to use as the target image for each iteration. This ensured that the largest jittering size was approximately one degree viewing angle.

To analyze the size of the dataset, we trained the reconstruction model with a variable number of training samples for 1,000 epochs with a batch size of 60. The rest of the hyperparameters were the same as in the previous analysis.

### Dataset From Shen et al. (2019)

We used an fMRI dataset from our previous reconstruction study (Shen et al., 2019). This dataset was used to reconstruct stimulus images from the visual features of a deep convolutional neural network that was decoded from the brain. The dataset analyzed for this study can be found in the OpenNeuro (https://openneuro.org/datasets/ds001506) repository.

The dataset comprises fMRI data from three human subjects. For each subject, the stimulus images in the dataset are categorized into four types: training and test natural images, artificial shapes, and alphabetical letters. The natural images used for the experiment were selected from 200 representative object categories in the ImageNet dataset (2011, fall release) (Deng et al., 2009). The training dataset of natural images were 1,200 images that were taken from 150 object categories and the test dataset of natural images were 50 images from the remaining 50 object categories. Thus, the categories used in the training and test datasets did not overlap. The artificial shapes were 40 images obtained by combining 8 colors and 5 shapes. The artificial shapes stimuli set was controlled by shape and color, but figure-ground separation and brightness were consistent across all the stimuli. The alphabetical letters were 10 black letters from the English alphabet. The alphabetical letters stimuli set had consistent color, brightness and figure ground separation. The only variable in this stimuli set was the shape of the alphabet.

The image presentation experiments comprised four distinct types of sessions that corresponded to the four categories of stimulus images described above. In one training-session set (natural images), 1,200 images were each presented once. This set of training session was repeated five times. In each test-session (natural image, artificial shape, and alphabetical letters), 50, 40, and 10 images were presented 24, 20, and 12 times each, respectively. The presentation order of the images was randomized across runs.

The fMRI data obtained during the image presentation experiment were preprocessed for motion correction followed by co-registration to the within-session high-resolution anatomical images of the same slices and subsequently to T1-weighted anatomical images. The coregistered data were then re-interpolated as 2 × 2 × 2 mm voxels.

The fMRI data samples were created by first regressing out nuisance parameters from each voxel's amplitude for each run, including a linear trend and temporal components proportional to six motion parameters. These were calculated by the SPM (http://www.fil.ion.ucl.ac.uk/spm) motion correction procedure. After that, voxel amplitudes were normalized relative to the mean amplitude of the initial 24 s rest period of each run, and were despiked to reduce extreme values (beyond ± 3 SD for each run). The voxel amplitudes were then averaged within each 8 s (training sessions) or 12 s (test sessions) stimulus block (four or six volumes), after shifting the data by 4 s (two volumes) to compensate for hemodynamic delays.

The voxels used for reconstruction were selected from the VC, which consisted of lower-order visual areas (V1, V2, V3, and V4) as well as higher-order visual areas (the lateral occipital complex, fusiform face area, and parahippocampal place area). The lower-order regions were identified using retinotopy experiments and the higher-order areas were identified using functional localizer experiments (Shen et al., 2019).

The fMRI data from the training image dataset were further normalized to have zero mean and unit standard deviation for each voxel. The mean and standard deviation of the training fMRI data were then used to normalize the test fMRI data.

We performed trial-averaging for the test fMRI data while we considered each trial as an individual sample for the training fMRI data. Therefore, to compensate for the statistical difference between training and test fMRI data, we rescaled the test fMRI data by a factor of $\sqrt{n}$ where *n* is number of trials averaged, before we use the test fMRI data as the input to the generator.

We train reconstruction models with the training natural images and their corresponding fMRI data for each individual subject, and test reconstruction models with the test fMRI dataset of the corresponding subject. For training in the dataset size-analysis, we initially selected a fixed number of training images and their corresponding fMRI data from five trials. As we increased the size of the dataset, we added more training images and fMRI data. Specifically, we gradually increased the size of the training dataset from 120 (5 × 24) to 6,000 (5 × 1,200) training samples.

### Evaluation

We evaluated the quality of reconstruction using both objective and subjective assessment methods. For both methods, we performed a pairwise similarity comparison, following previous studies (Cowen et al., 2014; Lee and Kuhl, 2016; Seeliger et al., 2018; Shen et al., 2019), in which one reconstructed image was compared with two candidate images: the original stimulus image from which the reconstruction was derived and a “lure” image, which was a different test image. The lure image was randomly selected from the test dataset of the same type as the original stimulus image. For each reconstructed image, the pairwise similarity comparison was conducted for all possible combinations of candidate images: the original stimulus image and every other stimulus image of the same type in the test dataset. For example, to evaluate the reconstruction quality for one of the 50 test natural images, the lure image is randomly selected from the remaining 49 test natural images. Then, for each reconstructed natural image, the pairwise similarity comparison is conducted for all 49 pairs of candidate images.

For the subjective assessment, we conducted a behavioral experiment similar to Shen et al. (2019). In this experiment, a group of 13 raters (6 females and 7 males, aged between 19 and 48 years) were presented with a reconstructed image and two candidate images and were asked to select the candidate image that appeared more similar to the reconstructed image. The trials for different test images were presented in a randomized order for each rater to prevent them from memorizing the correspondence between reconstructed and the true images.

For the objective assessment, we conducted pairwise similarity comparison analysis based on two metrics separately: Pearson correlation coefficient and structural similarity index (SSIM) (Wang et al., 2004). We computed the two metrics between the reconstructed image and each of the two candidate images. For the pairwise similarity comparison, we selected the candidate image with the higher Pearson correlation coefficient or higher SSIM, respectively.

For computing pixel-wise Pearson correlation coefficients, we first reshaped an image (a 3D array with dimensions of height, width, and RGB color channels) into a 1-dimensional vector. During this reshaping, the pixels of different color channels are concatenated in a vector. Then we calculated the Pearson correlation coefficient between the reshaped reconstructed and candidate images.

Since Pearson correlation coefficient considers each pixel as an independent variable, we also used SSIM to take into account the similarity of local structures of the spatially close pixels between two given images. We computed SSIM between the reconstructed and candidate images in the original 2D form for each of the RGB color channels, and then average the SSIM across the RGB color channels.

For both assessments, we calculated the percentage of trials in which the original stimulus image was selected, and used this value as the reconstruction accuracy of each reconstructed image. Trials for each reconstructed image were conducted by pairing the original stimulus image with every other stimulus image of the same type. For the study of dataset size, we reduce the trials for each reconstructed image by randomly selected 500 trials (10 trials for each test image) from all the possible trials, while the selected trials are fixed for all the conditions (here the modes trained with different number of samples) to be compared. For each type of test images (natural images, artificial shapes and alphabetical letters), we used the mean reconstruction accuracy as the quality measure, which was obtained by averaging across all the samples after pooling the three subjects.

We compliment the evaluation using pairwise similarity comparison with modified RV coefficient (Smilde et al., 2009). We compute the modified RV coefficient between two matrices: matrix of the reconstructed images and matrix of the true images. The rows of both these matrices correspond to test samples and columns correspond to individual pixels. With this setting, the modified RV coefficient evaluates the correlation between similarity relation within the true images and within the reconstructed images. We compared the results with a baseline of modified RV coefficient computed with randomly shuffled ordered of reconstructed images and correctly ordered true images to see whether the reconstructions preserve the similarity relation among the true images.

We conducted another behavioral experiment to study the effect of different loss terms in the proposed approach. Another group of 5 raters (2 females and 3 males, aged between 25 and 37 years) were presented with one original stimulus image and two reconstructed images that were generated from different combinations of loss terms. The raters were asked to judge which of the reconstructions more resembled the original stimulus image. This pairwise comparison was conducted for 6 pairs of loss term combinations for each stimulus image in the test dataset. We used the winning percentage as the quantitative measure for comparing reconstructions that were generated using different combinations of loss terms. The winning percentage was the percentage of trials in which the reconstruction from one combination was judged better than that of the other. For computing the winning percentage from objective metrics, the reconstructions with higher similarity (Pearson correlation coefficients or SSIM) were selected. For more details regarding the design of the behavioral experiments, please refer to Shen et al. (2019).

## Results

### Image Reconstruction

We trained the reconstruction model on the Shen et al. (2019) training-session samples of fMRI visual perception data. In the training session, each stimulus image had been presented to each subject five times. Here, we treated each stimulus presentation as a separate training sample for the reconstruction model. Therefore, the training dataset we used consisted of 6,000 (5 × 1,200) samples.

**Figure 2:**
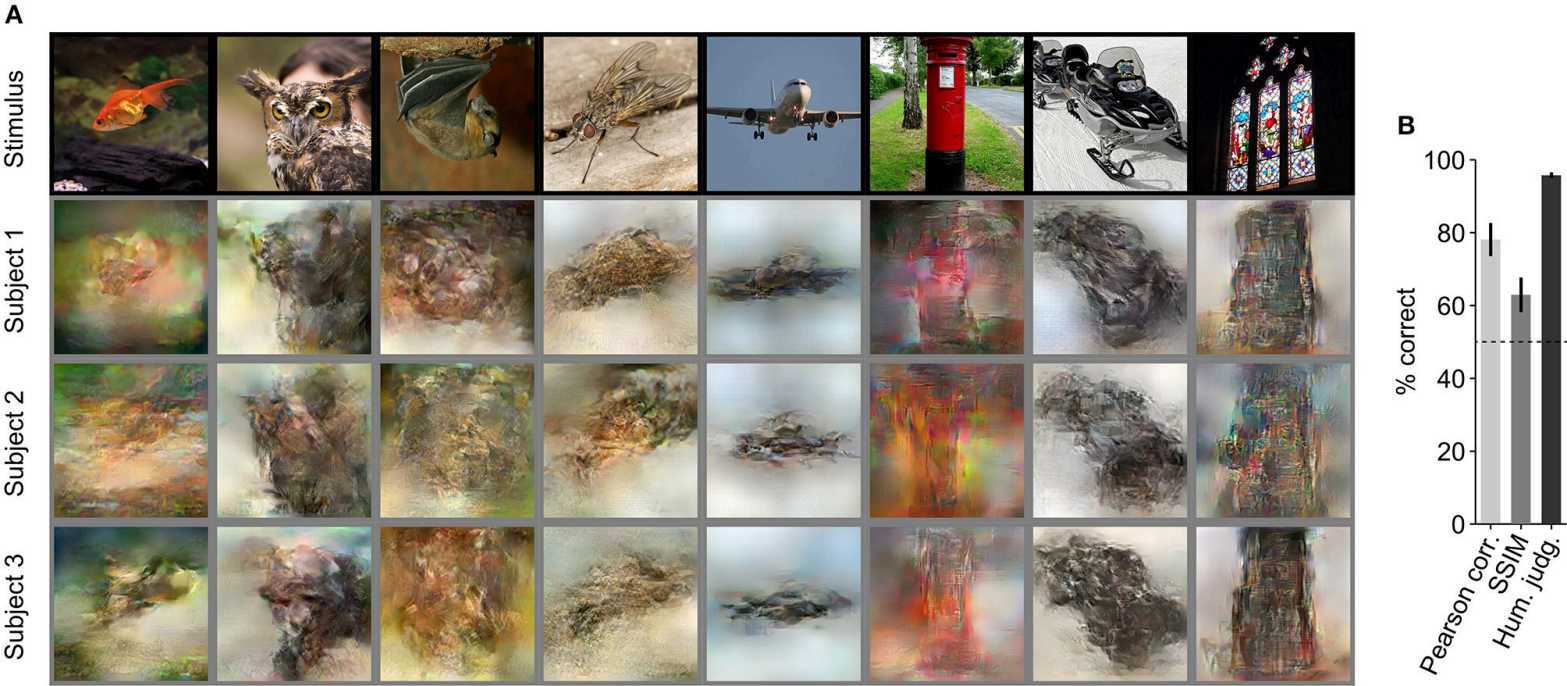
Reconstruction of natural images. **(A)** Stimulus and reconstructed natural images. The stimulus images (in black frames) are shown in the top row. Three corresponding reconstructed images (in gray frames) from each of the three subjects are shown underneath. **(B)** Reconstruction accuracy for natural images in terms of the accuracy of pairwise similarity comparison based on Pearson correlation, structural similarity index (SSIM) and human judgment (error bars, 95% confidence interval (CI) across samples; three subjects pooled, the number of samples (*N*) = 150; chance level, 50%).

We evaluated reconstruction quality using three test datasets: natural images, artificial shapes and alphabetical letters. For generating reconstructions, fMRI samples corresponding to the same image (24 samples for the natural image session, 20 for the artificial shapes session, and 12 for the alphabetical letters session) were averaged across trials to increase the signal to noise ratio. The averaged fMRI samples were used as input to the trained generator (Figure 1B). Figure 2A shows example images from the natural image test dataset and their corresponding reconstructions from three different subjects. The reconstructions from all three subjects closely resembled shape of the object in the natural image stimuli. The color, however, was not preserved in some of the reconstructions. The reconstruction results from our model show that despite utilizing a small dataset, training a model from scratch and reconstructing visually similar images from fMRI data was possible with high accuracy (Figure 2B) The mean reconstruction accuracy (three subjects pooled, *N* = 150) is 78.1% by Pearson correlation (78.9, 75.3, and 79.9% for Subject 1, 2, and 3), 62.9% by SSIM (63.0, 61.9, and 63.8% for Subject 1, 2, and 3), and 95.7% by human judgment (95.6, 95.1, and 96.4% for Subject 1, 2, and 3). Additionally, we calculated modified RV coefficient, which evaluates the correlation between the similarity relation within the true images and the reconstructed images to see whether the reconstructions preserve the similarity relation within the true images. The higher modified RV coefficients (0.34, 0.32, and 0.32 for Subject 1, 2, and 3) for natural image test dataset as compared to the baseline calculated by random permutation (*p* < 0.0001 for all three subjects, permutation test) demonstrate that reconstructed images from our approach preserve the similarity relation within the true images.

**Figure 3:**
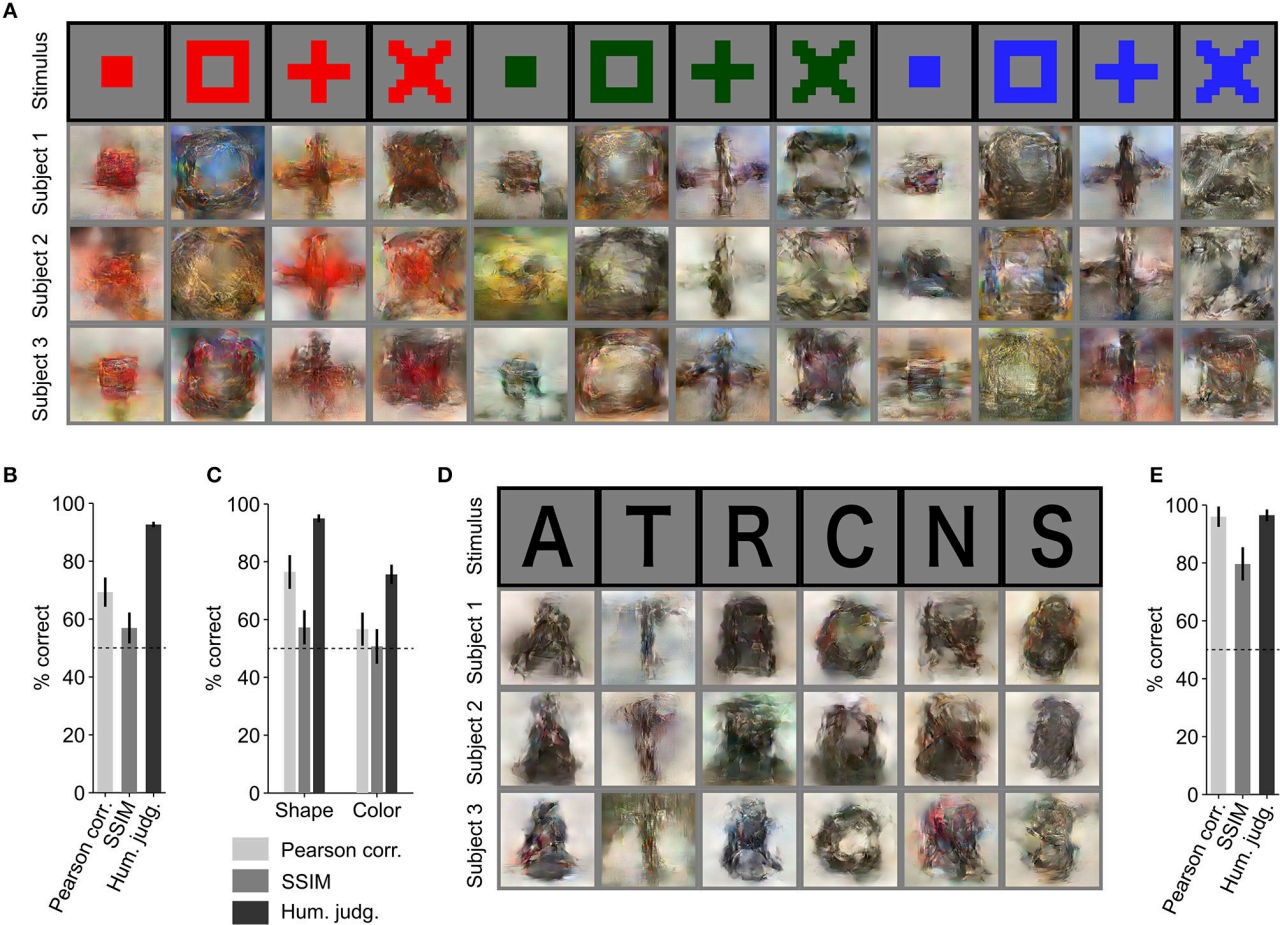
Reconstruction of artificial images. **(A)** Reconstruction of artificial shapes. The stimulus images (in black frames) are shown in the top row. Three corresponding reconstructed images (in gray frames) from each of the three subjects are shown underneath. **(B)** Reconstruction accuracy for artificial shapes. **(C)** Reconstruction accuracy for both shape and color. **(D)** Reconstruction of alphabetical letters. **(E)** Reconstruction accuracy for alphabetical letters. For **(B,C,E)**, reconstruction accuracy is assessed in terms of the accuracy of pairwise similarity comparison based on Pearson correlation, structural similarity index (SSIM) and human judgment (error bars, 95% CI across samples; three subjects pooled, *N* = 120 for artificial shapes, *N* = 30 for alphabetical letters; chance level, 50%).

Further, we evaluated the generalizability of our reconstruction model (trained solely with natural images and fMRI responses) using artificial images as similarly performed by Shen et al. (2019) (Figure 3A). Using the proposed approach, artificial shapes were reconstructed with high accuracy (Figure 3B. 69.3% by Pearson correlation, 56.9% by SSIM, and 92.7% by human judgment) and alphabetical letters were also reconstructed with high accuracy (Figures 3D,E; 95.9% by Pearson correlation, 79.6% by SSIM, and 96.4% by human judgment), even though the model was trained on natural images.

From the results for artificial shape reconstruction, we observed that the shape of the stimulus was well preserved in the reconstructions. However, the color was preserved only for the red-colored shapes. To evaluate reconstruction quality in terms of shape and color, we compared reconstructed images of the same colors and shapes, respectively. The quantitative results are shown in Figure 3C (shape: 76.5% by Pearson correlation, 57.3% by SSIM, and 95.0% by human judgment; color: 56.7% by Pearson correlation, 50.7% by SSIM, and 75.6% by human judgment) and confirm that the reconstructed images were more similar in shape to the original images than in color.

While the main purpose of this study is to evaluate the potential of the end-to-end method in learning direct mapping from fMRI data to visual images, we compared the reconstruction accuracy of the proposed method with that of Shen et al. (2019) to analyze the difference between the two methods. We observed that our new method achieved almost same performance as Shen et al. (2019) on the Pearson correlation metric (natural images: ours 78.1 vs. 76.1%; two-sided signed-rank test, no significantly difference, *N* = 150), whereas our new method did not outperform Shen et al. (2019) on the subjective judgment (natural images: ours 95.7 vs. 99.1%; two-sided signed-rank test, *P* < 0.006, *N* = 150). Shen et al. (2019) used a natural image prior that helps their reconstructions look more natural, which could explain why that method outperforms our new method in terms of human judgment. We tried to introduce a natural image prior through use of a discriminator, but the reconstructions did not appear as natural as those from Shen et al. (2019).

### Effect of Dataset Size

The results of the previous analyses show that it is possible to reconstruct images from human brain activity by training an end-to-end model from scratch with only 6,000 training samples. Next, we sought to investigate the effect of dataset size on reconstruction quality. We checked how many samples are enough to achieve recognizable reconstruction and assessed the possibility of improving reconstruction quality using more training samples.

**Figure 4:**
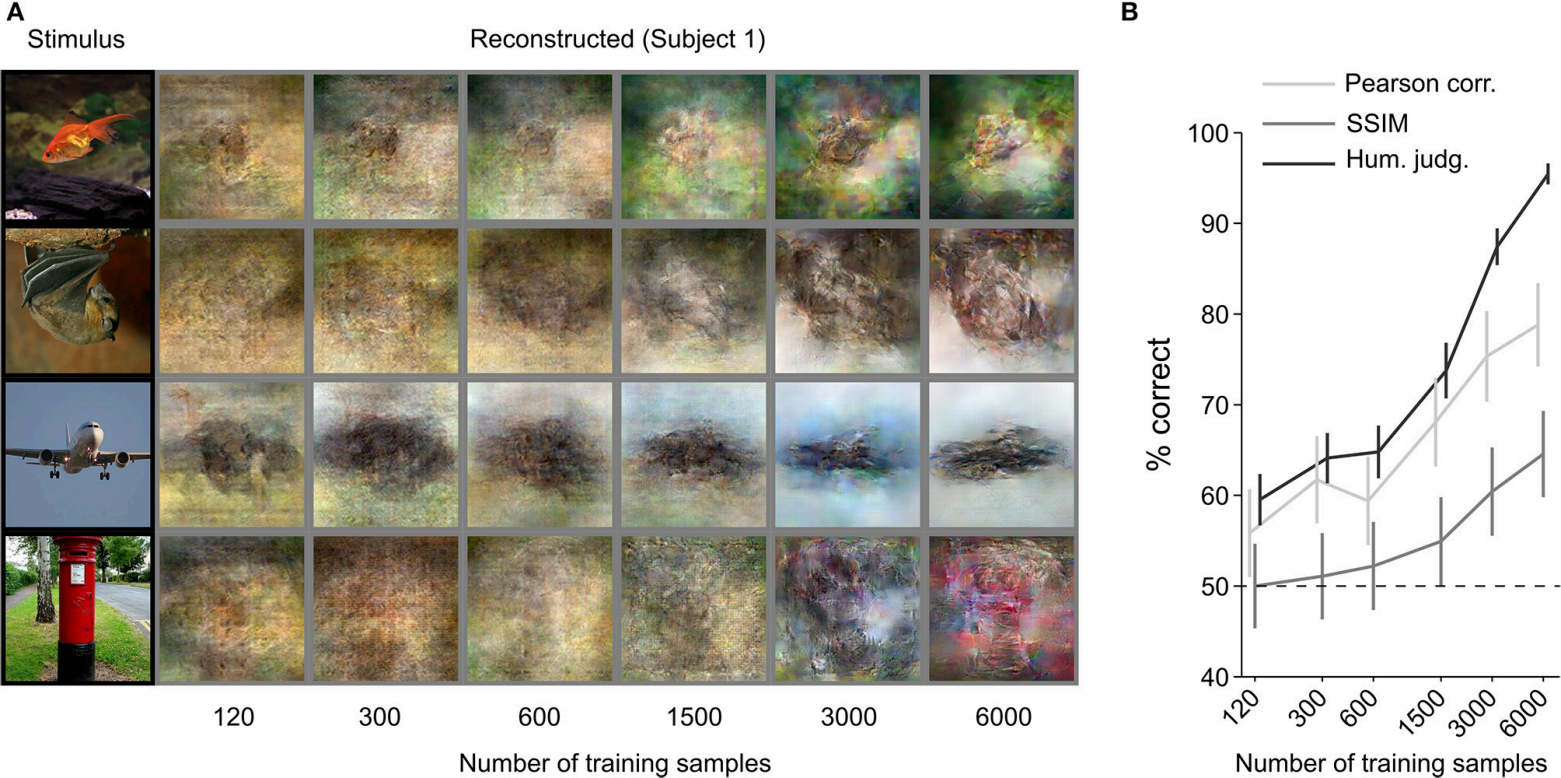
Effect of training-dataset size. **(A)** Reconstruction from brain activity (Subject 1) using models trained with different dataset sizes. The stimulus images (in black frames) are shown in the first column. The corresponding reconstructed images (in gray frames) are shown to the right of each stimulus image (from left to right, the number of training samples increases). **(B)** Reconstruction accuracy in terms of the accuracy of pairwise similarity comparison based on Pearson correlation, structural similarity index (SSIM) and human judgment (error bars, 95% CI across samples; three subjects pooled, *N* = 150, chance level, 50%). The horizontal axis is scaled using a base 2 logarithm.

We increased the training dataset from 120 to 6,000 (120, 300, 600, 1,500, 3,000, and 6,000) samples. Figure 4 shows a qualitative comparison of reconstructions (Figure 4A) and the quantitative objective and human judgment scores (Figure 4B). Through visual inspection of the reconstruction results in Figure 4A, we can see that reconstruction quality improved with the number of training samples. Objective and human judgment scores quantitatively confirm this trend. The results showed that the increasing trend in the reconstruction quality is not saturated for our reconstruction model, which suggests that although we can obtain highly accurate reconstructions with only 6,000 training samples, better reconstruction quality might be achieved if larger datasets are available.

### Effect of Loss Functions: Ablation Study

**Figure 5:**
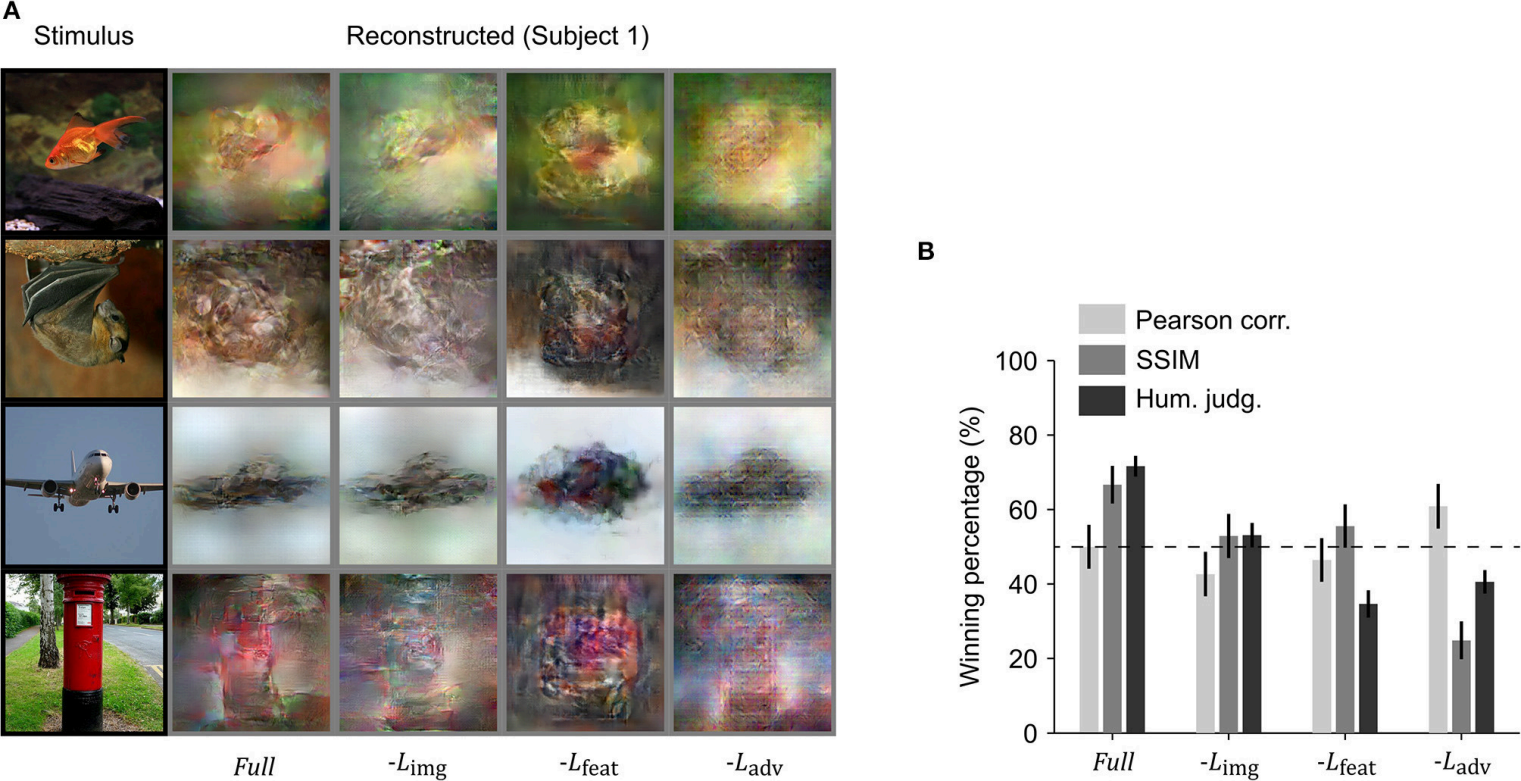
Ablation of loss terms. **(A)** Reconstruction from brain activity (Subject 1) using reconstruction models with some loss components removed. The stimulus images (in black frames) are shown in the first column. The corresponding reconstructed images (in gray frames) obtained with different models are shown to the right of each stimulus image (from right to left, the model is: full reconstruction model (*Full*), with image loss removed (−*L*_img_), with feature loss removed (−*L*_feat_), and with adversarial loss removed (−*L*_adv_). **(B)** Reconstruction accuracy in terms of winning percentage of pairwise similarity comparison based on Pearson correlation, structural similarity index (SSIM) and human judgment (error bars, 95% CI across samples; three subjects pooled, *N* = 150, chance level, 50%). The winning percentage is the percentage of pairwise similarity comparison trials in which the reconstruction from one model was judged better than that of the other.

We performed an ablation study to understand the effects of the different loss functions used in training the reconstruction model. We removed one loss function at a time and compared the reconstructions with those obtained using all three loss functions. Visual inspection showed that the best resemblance to the original images was obtained using all three loss terms (Figure 5A). To quantitatively compare the reconstruction quality of different models in the ablation study, the winning percentage of the pairwise similarity comparisons based on either objective or human judgment was used. The difference in winning percentage between the model optimized with all three loss terms and the model optimized with one loss term removed indicates the importance of the corresponding loss term. From Figure 5B, we can observe that the model trained with all three loss terms showed the highest winning percentage followed by the model where the loss in the image space is removed. The results demonstrate that the model trained with all three loss terms was preferred by the human raters.

Removing the loss in image space resulted in a moderate drop for both objective and subjective assessments (Pearson correlation 7.3% decrease, SSIM 13.8% decrease, and human judgment 18.5% decrease), but the difference in human judgement was not as pronounced as it was for the other two loss functions. Removing feature loss produced the highest drop in winning percentage for human judgment (36.9% decrease) and a moderate drop in Pearson correlation (5.6% decrease) and SSIM (11.1% decrease). This demonstrates the importance of optimization in high dimensional feature space, as it not only enhances the spatial details, but also makes the reconstruction more perceptually similar to its corresponding original stimulus image. Although removing adversarial dramatically reduced human judgement scores (30.0% decrease) and SSIM (41.8% decrease), it surprisingly showed improvement in Pearson correlation (10.9% increase). This suggests that optimizing adversarial loss forces the reconstruction to appear closer to a natural image distribution and preserve structural similarity but has a negative impact on preservation of the spatial details.

## Discussion

Here, we have demonstrated that end-to-end training of a DNN model can directly map fMRI activity in the visual cortex to stimuli observed during perception, and thus reconstruct perceived images from fMRI data. The reconstructions of natural images were highly similar to the perceived stimuli in shape, and in some cases in color (Figure 2). Although trained only on natural images, the model generated accurate reconstructions of artificial shapes and alphabetical letters (Figure 3), thus showing generalizability that is similar to Shen et al. (2019). We also demonstrated that reconstruction quality improved as the number of training samples increased (Figure 4), and thus we may be able to further improve reconstruction accuracy with even more training samples.

We performed an ablation study by removing one loss function at a time to understand the importance of each loss term used for training the proposed model (Figure 5). The results showed that the model trained with all three loss terms achieved the best performance in terms of human judgement while the model trained without the adversarial loss showed the best performance in terms of Pearson correlation. The removal of loss in image space resulted in moderate changes in winning percentage calculated from behavioral experiments and both objective measures (Pearson correlation and SSIM). The removal of feature loss resulted in a drop in all the three types of winning percentage, although the drop in human ratings was more pronounced. Although removal of adversarial loss showed significant increase in winning percentage based on Pearson correlation, winning percentage based on human ratings and SSIM dropped significantly. This suggests that the addition of adversarial loss in the optimization process constrains the reconstructed images so that their distribution is closer to that of the training images (natural images). The increase in Pearson correlation winning percentage, however, suggests that adversarial loss has negative impact on preserving the spatial details of the reconstructed image. The results suggest that both the perceptual and adversarial losses are critical for our end-to-end deep image reconstruction model to achieve perceptually similar reconstructions.

Earlier studies on decoding stimuli in pixel space either searched for a match in the exemplar set (Naselaris et al., 2009; Nishimoto et al., 2011) or tried to reconstruct the stimulus (Miyawaki et al., 2008; Wen et al., 2016; Güçlütürk et al., 2017; Han et al., 2017; Seeliger et al., 2018; Shen et al., 2019). In the exemplar matching methods, visualization is limited to the samples in the exemplar set and hence these methods cannot be generalized to stimuli that are not included in the exemplar set. In contrast, reconstruction methods are more robust in generalizing to a new stimulus domain (Figure 3).

DNN-based reconstruction methods have typically avoided directly training a DNN model for reconstruction (Güçlütürk et al., 2017; Han et al., 2017; Seeliger et al., 2018; Shen et al., 2019). Instead, they have used decoded features as a proxy for hierarchical visual representations encoded in the fMRI activity that was used as the input to a reconstruction module. This method is effective since the decoded features can easily be plugged into known image reconstruction/generation methods. It is also thought to be efficient given the lack of large-scale diverse fMRI datasets (which contrasts with the large computer-vision datasets used for end-to-end training of vision tasks). The lack of large fMRI datasets makes learning a direct mapping from brain activity to stimulus space difficult without overfitting to the training dataset. Thus, developing a way to learn this direct mapping from limited numbers of training samples was the main motivation for this work.

A potential advantage of direct mapping is that it avoids information loss that occurs in the feature-decoding step. Even though the decoded features are correlated with the original image features, in Horikawa and Kamitani (2017) the maximum correlation coefficient on average was < 0.5. Thus, we argue that information in the decoded features is not all the visual information that can be decoded from the brain. Therefore, if enough training samples are available, direct mapping may help in preventing this information loss.

Our proposed method can easily be extended to other modalities such as text, sounds and video. This can be achieved by a suitable choice of generator, discriminator, and comparator modules for the corresponding modality. Further, our approach can be extended for reconstruction of multimodal data where a single generator module with multiple heads can generate reconstructions of multiple modalities simultaneously. Therefore, we believe an end-to-end approach has a wide potential for transforming the internal representations of the brain to meaningful visual and auditory contents for brain-machine interfaces.

## Ethics Statement

This study was carried out in accordance with the recommendations of the Ethics Committee of Advanced Telecommunications Research Institute International (ATR). The protocol was approved by the Ethics Committee of ATR. All subjects gave written informed consent in accordance with the Declaration of Helsinki.

## Author Contributions

YK directed the study. GS, KD, and KM developed the reconstruction methods. TH performed the experiments. GS performed the analyses. KD and YK wrote the paper.

### Conflict of Interest Statement

The authors declare that the research was conducted in the absence of any commercial or financial relationships that could be construed as a potential conflict of interest.
